# Decoding the Cryptic Proteome Between Antigens and Novel Functional Proteins

**DOI:** 10.1002/eji.70102

**Published:** 2025-12-19

**Authors:** Emma G. Bawden, Sebastian Amigorena, Yago A. Arribas

**Affiliations:** ^1^ Inserm U932 Immunity and Cancer Institut Curie PSL University Paris 75005 France

**Keywords:** antigen presentation, cryptic proteome, DRiPs, immunopeptidomics, ribosome profiling

## Abstract

The widespread translation of cryptic proteins derived from the non‐coding genome expands the complexity of the human proteome. A vast majority of cryptic proteins are expressed at low levels, rapidly degraded and efficiently presented on class I major histocompatibility complexes (MHC‐I). On the other hand, some cryptic proteins are stable and functional and may integrate into the proteome through ongoing selective pressures. Herein, we propose a model in which the translation of cryptic proteins increases the diversity of functional proteins on which evolution can act and, during this trial‐and‐error process, provides a valuable source of antigens for immunosurveillance.

## Graphical abstract

Unannotated splicing and translation of non‐canonical open reading frames generate a diverse repertoire of cryptic proteins. Most are rapidly degraded, rendering them possible MHC‐I substrates. Yet, a subpopulation can adopt stable structures and impart advantageous functions. This dichotomy both expands the antigen landscape and provides a framework for protein evolution.

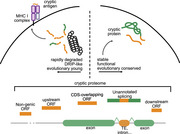

## 1 Introduction

**FIGURE 1:**
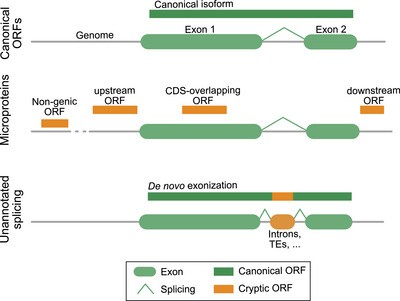
Types of cryptic proteins. Microproteins can originate from unannotated ORFs embedded either within a CDS, upstream or downstream ORFs, or from non‐genic regions. Cryptic proteins can result from unannotated splice sites leading to the de novo exonization of cryptic sequences, primarily from introns.

The Human Genome Project generated the first sequence of the entire human genome, enabling the annotation of protein‐coding genes. Databases containing these annotations, including GENCODE and UniProt, provide the current catalogue of human proteins, which continue to be updated. Existing annotations define what we consider the canonical proteome, but underestimate the full complexity of proteomic diversity. Protein isoforms, unreviewed proteins and the translation of novel open reading frames (ORFs) largely expand the human proteome beyond the limits of any annotation. Advances in mass spectrometry (MS)‐based proteomics and ribosome profiling (RiboSeq), which sequences ribosome‐protected mRNA fragments, have revealed the widespread translation of proteins that partially or fully derive from the non‐coding genome, referred to as the cryptic proteome [1, 2, 3, 4]. Cryptic proteins can be grouped into two main types: microproteins (typically < 100 amino acids) that are transcribed from short ORFs and cryptic isoforms that arise from unannotated alternative splicing (Figure 1). Microproteins are commonly derived from intergenic regions, including non‐protein‐coding transcripts such as long non‐coding RNAs (lncRNA) or transposable elements (TEs). They can also be transcribed from unannotated ORFs embedded within protein‐coding genes [3]. Cryptic isoforms that result from the splicing of non‐coding regions with known exons commonly incorporate intronic sequences [5, 6], including TEs [7, 8, 9, 10].

**FIGURE 2:**
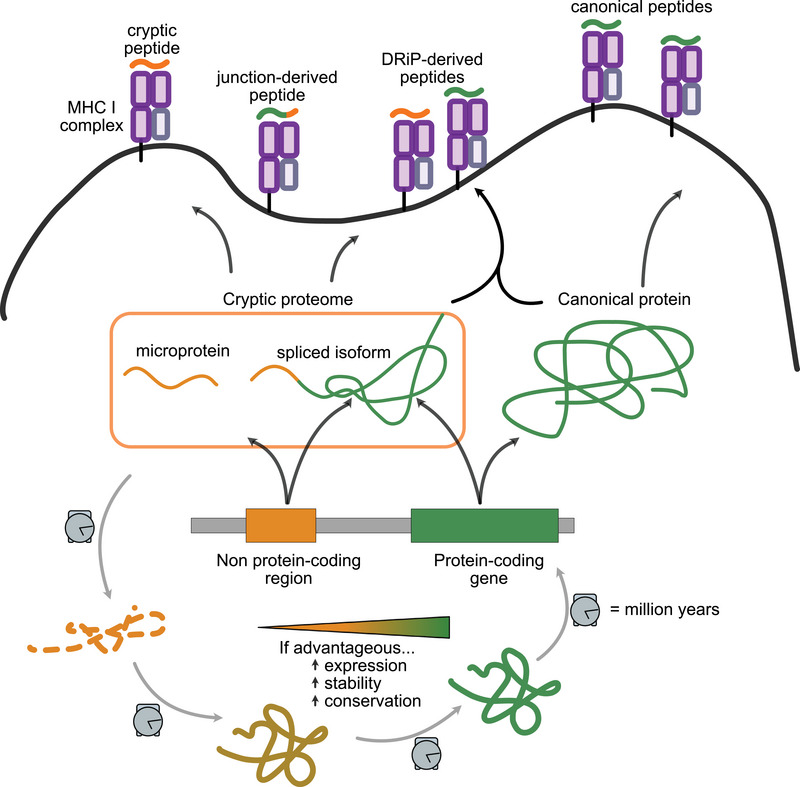
Distinct cryptic proteins populate the immunopeptidome and the whole‐cell proteome. Like DRiPs, some cryptic proteins may be unstable, readily degraded and directed to the MHC‐I loading pathway. Meanwhile, a subset of cryptic proteins may exhibit secondary structure and function that, if advantageous, render it stable in the genome and conserved in evolution.

A central question is whether these cryptic proteins represent functional entities or are predominantly short‐lived products of aberrant translation (Figure 2). The latter may be analogous to the previously described defective ribosomal products (DRiPs), which are polypeptides that arise from inefficient translation, undergo rapid degradation, and are readily detected in the MHC‐I immunopeptidome [11, 12]. This review seeks to explore the properties and functions of cryptic proteins and propose a model to integrate the non‐mutually exclusive hypotheses that the cryptic proteome can (i) constitute translational mistakes with non‐functional protein products, (ii) provide antigens for immune surveillance and (iii) encode a subset of functional proteins subject to selective pressures.

## 2 Cryptic Proteins Are Enriched in the Immunopeptidome Compared to the Whole‐Cell Proteome

Whilst transcription of ‘non‐coding’ DNA is readily detectable by conventional RNA sequencing, cryptic protein discovery requires evidence of translation [1]. As such, both RiboSeq and MS‐based sequencing of MHC‐I presented peptides (immunopeptidomics) have been instrumental in identifying cryptic proteins. In contrast, cryptic proteins are far less detectable by MS in whole‐cell proteomics. For example, we identified approximately 10 times more TE‐containing isoforms using RiboSeq than with deep whole‐cell proteomics [8]. Similarly, in a study by Chen et al., from human induced pluripotent stem cells, around 6000 cryptic proteins were detected by RiboSeq, 240 were identified through immunopeptidomics and only 36 were found in whole‐cell proteomics [13].

The increased detection of cryptic proteins in the immunopeptidome could be explained by both technical and biological reasons. First, the shorter length of cryptic proteins compared to canonical proteins can impact their detection by whole‐cell proteomics. While canonical proteins have a mean length of around 600 amino acids and are encoded by 14 exons on average [8], microproteins are often encoded by just one or two exons and average 49 amino acids in length [12]. Unannotated isoforms often result in truncated proteins with an average length of 90 amino acids [8]. Since whole‐cell proteomics relies on the digestion of proteins into peptides, usually by trypsin, which cleaves at arginine and lysine residues, shorter proteins have a reduced number of possible cleavage sites. This may obfuscate their detection as reliable identifications. In addition, trypsin digestion poses limitations on the identification of cryptic peptides derived from splicing junctions because exon boundaries exhibit high usage of arginine and lysine [14].

On the other hand, the enrichment of cryptic proteins in the immunopeptidome could be attributed to their higher degree of intrinsic disorder and lower predicted stability compared to canonical proteins, directing them more readily to degradation and MHC‐I‐loading [8, 12, 15]. Similarly, cryptic proteins can contain premature stop codons, targeting them for degradation by nonsense‐mediated mRNA decay, an RNA quality control pathway [16]. Overall, cryptic proteins represent a small fraction of the whole protein content of the cell and are therefore difficult to identify by whole‐cell proteomics. In contrast, HLA‐I presentation does not rely on protein abundance but on protein degradation, which could explain why cryptic proteins represent a source of endogenous antigenic peptides.

## 3 The Overlap Between the Cryptic Immunopeptidome and DRiPs

CD8^+^ and CD4^+^ T cells effectuate immune responses upon recognition of their cognate antigen presented on MHC‐I and MHC‐II (HLA‐I and HLA‐II, in humans), respectively. Antigen presentation relies on the degradation of polypeptides or proteins into short peptides that can efficiently bind MHC‐I molecules. Whilst some cells can take up and process exogenous material through the HLA pathway, most HLA‐bound peptides are derived from intracellular origins. However, the precise sources of intracellular antigens remain incompletely defined. HLA‐I‐presented peptides can derive either from the natural turnover of cellular proteins (*proteome model*) or from newly synthesized DRiPs (*DRiP hypothesis*) [11, 17]. Indeed, the possibility of both stable and unstable proteins being efficiently presented by HLA‐I molecules broadens the potential antigenic landscape. DRiPs originate from mutated, aberrantly translated or misfolded proteins and are sent for rapid proteasomal degradation (i.e., half‐life < 10 min, [18, 19, 20, 21]). The DRiP hypothesis was founded on the observation that infected cells can rapidly generate viral antigenic peptides from otherwise stable viral proteins. These viral proteins constitute a very low fraction of the total proteome and are recycled by turnover at low rates, if at all [11]. Therefore, the antigen presentation of rapidly degraded viral products may confer an evolutionary advantage by providing the host with rapid sensitivity to infection. For example, an immunogenic Influenza A viral peptide translated through an alternative reading frame of a downstream start codon of the NS1 gene was expressed at higher levels and presented 2–3 times faster than the immunodominant NP_147–155_ epitope [22]. Similarly, time‐course immunopeptidomics of SARS‐CoV‐2‐infected cells showed that viral peptides were detectable at 3 h and peaked at 6 h post‐infection [23]. Among these rapidly presented SARS‐CoV‐2‐derived peptides, approximately 25% were derived from cryptic ORFs. In addition, viral proteins presented within the first 3 h of infection were recognized by CD8^+^ T cells from COVID‐19 patients, which supports the role of DRiPs in the prompt immune surveillance of infected cells.

DRiPs are not only relevant for pathogen‐derived antigen presentation but also constitute a major source of endogenous HLA‐I peptides. Studies using stable isotope labelling with amino acids in cell culture (SILAC) to track the presentation of newly synthesized proteins reported that between 27% and 70% of the immunopeptidome derives from proteins degraded before their full maturation [18, 24]. A substantial portion of the cryptic proteome shares the key features of DRiPs; low stability and enrichment in the immunopeptidome. Yet, it remains unclear what fraction of cryptic proteins are rapidly degraded products versus long‐lived species. Nevertheless, cryptic proteins are efficiently presented on HLA‐I molecules at the cell surface, potentially rendering them an important source of antigens.

## 4 Cryptic Proteins as Targetable Cancer Antigens?

Cryptic proteins have recently emerged as exciting targets for antigen‐based therapies. Laumont et al. reported that around 6.5%–10% of HLA‐I‐associated peptides in a B‐lymphoblastoid cell line originate from cryptic translation [25], consistent with other proteogenomic studies across several tissues [26, 27, 28]. Whilst a large proportion of cryptic proteins are sample‐specific, a subpopulation is shared between patients and demonstrates varying levels of tumour‐enriched expression across several cancer types (e.g., in ovarian [29], breast [30], liver [31], colon [32], acute myeloid leukaemia [33] and lung [7]). In addition, some cryptic transcripts exhibit pan‐cancer expression, that is, they are enriched in many cancer types relative to healthy tissues [34, 35]. Increased translation of cryptic proteins in cancer can be attributed to oncogenic‐associated dysregulation of transcription and alternative splicing [36]. For example, mutations in the splicing factor *SF3B1* generate immunogenic neoantigens recognized by CD8^+^ T cells in at least 20% of *SF3B1*‐mutated uveal melanoma tumours [37].

Different types of cryptic proteins have been identified in cancer immunopeptidomes [27, 28, 38, 39] including translation from lncRNA [40], pseudogenes [28], circular RNAs [41] and TEs [42, 43, 44], as well as those derived from aberrant splicing events [45] including retained introns [46] or junctions between exons and transposable elements (JETs) [7, 9, 10]. Chong et al. developed a pipeline to accurately identify cryptic HLA‐I‐presented peptides, including lncRNA‐ and TE‐derived epitopes, and determined that 23% of the identified cryptic HLA‐I peptides were absent in normal tissue from the Genotype‐Tissue Expression (GTEx) database [28]. Several studies demonstrated that cryptic antigens can be immunogenic *ex vivo* [7, 47]. In ovarian cancer, ∼70% of the prioritized cryptic peptides were recognized by autologous T cells, which was superior to the other major classes of known tumour antigens. Supporting immunogenicity within the cryptic proteome, mice vaccinated with tumour‐specific HLA‐I‐restricted cryptic peptides exhibited delayed tumour growth [10, 48].

However, the immunogenicity of cryptic antigens *in vivo* requires further investigation. How their expression in healthy tissues affects immunogenicity, and whether they are expressed in the thymus during negative selection, are important questions to address. In addition, how the biochemical properties of different cryptic proteins affect their immunogenicity should be considered. For instance, can unstable cryptic proteins and DRiPs expressed by cancer cells efficiently prime an immune response? Unless T cells recognize these cryptic antigens through cross‐reactivity (i.e., viral mimicry), they would require de novo priming involving internalization by a migratory dendritic cell and subsequently cross‐presentation in secondary lymphoid organs. Whether unstable proteins are amenable to uptake, processing, and presentation by an antigen‐presenting cell (APC) remains to be established. In line with this, CD4^+^ T cells most commonly recognize cancer antigens in the tumour microenvironment through indirect display on professional APCs, consistent with the fact that many cancers do not express MHC‐II [49]. Whether cryptic proteins constitute a class of tumour antigens presented on MHC‐II for recognition by CD4^+^ T cells remains to be determined.

In addition, if unstable proteins and DRiPs do not confer important cellular functions, they could theoretically be downregulated by a cancer cell, leading to acquired resistance, were this antigen to be the target of therapy. Therefore, one could predict that if therapy were to target cryptic antigens, it would be done so in combination with other antigens, that is, personalized mutations or known tumour‐associated antigens. Targeting cryptic antigens may be especially relevant in tumours with low mutational burdens, where the number of antigen candidates from the canonical proteome is limited. The possibility of harnessing cryptic proteins in immunotherapy has evoked excitement in the field of oncoimmunology, but these aforementioned questions regarding antigenicity will need further exploration.

## 5 Distinct Cryptic Protein Populations Are Found in Whole‐Cell Proteome and the Immunopeptidome

Although cryptic proteins are more readily identified in the HLA‐I immunopeptidome, several have been identified in the whole‐cell proteome. Ruiz‐Cuevas et al. enriched for low‐molecular‐weight proteins (< 10 kDa) and found that cryptic proteins constituted around 80% of the MS‐identified proteins in this mass range [12]. Furthermore, there is restricted overlap between the cryptic proteins detected in the immunopeptidome and the total proteome, with reports ranging from just 6% [12] to as low as 0.8% shared proteins between both compartments [15]. The distinct subpopulations of cryptic proteins existing between the whole‐cell proteome and immunopeptidome could be attributed to their divergent properties; stable and functional proteins are more likely to be detected by whole‐cell proteomics and potentially rapidly degraded proteins are susceptible to enter the HLA‐I processing pathway.

In addition, the types of cryptic proteins differ between the two compartments (Table 1). While lncRNA‐derived peptides are abundant in both, the immunopeptidome is enriched in uORF‐derived peptides [15, 26] and the total proteome shows a higher prevalence of peptides mapping to pseudogenes (i.e., non‐functional gene copies). In addition, distinct proportions of TE classes observed among TE‐containing isoforms diverge between the immunopeptidome and the total proteome. JETs involving short interspersed nuclear elements (SINEs) are more prevalent in the MHC‐I immunopeptidome, while the exonization of long interspersed nuclear elements (LINEs) is enriched in the whole‐cell proteome [7, 8]. Translation of canonical proteins is generally initiated at an AUG start codon, while cryptic proteins show higher usage of non‐AUG start codons [12, 13, 15]. However, non‐AUG start codons (mostly CUG) are more enriched in cryptic proteins from the total proteome relative to those found in the immunopeptidome [12]. This points towards non‐canonical translation mechanisms specific to cryptic proteins. Could there exist a subset of ribosomes—crypto‐ribosomes—dedicated to (or at least more efficient in) the translation of cryptic ORFs, analogous to the previously proposed immunoribosomes involved in antigen presentation [50, 51]?

**TABLE 1:** Characteristics of cryptic proteins in the whole‐cell proteome and the immunopeptidome.

	Whole‐cell proteome	Immunopeptidome
Detection method	RiboSeq and whole‐cell proteomics	RiboSeq and MS‐based immunopeptidomics
Abundance	Scarce detection by total proteomics [8, 12, 13]	1%–15% of the total immunopeptidome [25, 26]
Protein stability	Similar levels to canonical proteins [8]	High instability (i.e., DRiPs) [12]
Start codon usage	Similar rates of AUG and CUG [12]	Higher usage of AUG, but less than canonical proteins [12]
Microproteins	Mainly derived from pseudogenes and lncRNAs [15]	uORF and lncRNAs [15, 26, 28, 31]
JET‐derived isoforms	Enriched in LINEs [8]	Enriched in SINEs [7, 10]

## 6 Cryptic Proteins Can Be Functional

Cryptic proteins are heterogeneous in nature, and some exhibit a clear biological relevance [13, 52, 53]. Some cryptic ORFs are expressed in response to cellular stress and may produce proteins that, when misfolded, induce cytotoxic endoplasmic reticulum stress and apoptosis [54]. Nonetheless, cryptic proteins can also be functionally relevant under physiological conditions [55, 56]. CRISPR‐Cas9 screening of microproteins revealed that several cryptic ORFs are essential for cell survival, with some studies reporting that 20% of the targeted microproteins were indispensable [13, 57, 58, 59, 60]. The structural flexibility of microproteins enables interaction with the canonical proteome, often the closest proximal canonical coding sequence (CDS). For example, uORFs‐derived microproteins of HAUS6 and MIEF1 interact with their respective canonical isoforms encoded in the downstream CDS [13]. The uORF‐derived HAUS6 microprotein localizes to the centrosome and its deletion induces cell cycle arrest, in line with the function of the canonical HAUS6 protein, which is involved in microtubule organization. Similarly, like the canonical MIEF1 protein, the uORF‐derived MIEF1 microprotein localizes to the mitochondria and regulates mitochondrial fission. On the other hand, SNSD1 uORF, which is associated with *MYCN* amplification, is upregulated independently of its canonical counterpart in medulloblastoma and supports tumour cell survival [57]. LncRNAs can also form functional microproteins. For example, GT3‐INCP and SMIMP microproteins, encoded by primate‐specific lncRNAs, interact with the cellular machinery to promote tumour progression in breast and colon cancers [59, 61].

A subset of TE‐derived isoforms is recurrently expressed across individuals and datasets [7, 8]. Some of these isoforms are equally as stable as their corresponding canonical isoforms and exhibit defined subcellular localizations. Notably, TE exonization in tumour suppressor genes such as *PTEN* and *WWOX* leads to the production of protein isoforms with altered localization and function compared to their canonical counterparts [8]. Whereas the canonical PTEN protein is mainly present in the cytosol, the PTEN JET‐ORF contributes to histone regulation through enhanced nuclear localization. TE exonization in *IFNAR2* codes for a predominant isoform expressed in immune cells that acts as a decoy receptor to inhibit interferon signalling [62]. Similarly, exonization of an intronic LINE in *PD‐L1* leads to a soluble isoform, which acts as an antagonist of the canonical PD‐L1 [63].

Supporting their biological functions, cryptic proteins can adopt secondary structures. Exonized TEs are enriched in host proteins with high content of α‐helices and preferentially adopt an α‐helix conformation themselves [8]. Microproteins are also richer in α‐helices and depleted in β‐strands, compared to canonical proteins [64, 65, 66]. As the identification of cryptic ORFs steadily increases, the ability to characterize functions of novel proteins at a similar rate remains challenging. Determining the functions of cryptic proteins will reveal important aspects of human biology in health and disease.

## 7 Evolution Through the Cryptic Proteome

The appearance of novel ORFs is a multi‐step process in which a series of random mutations begets the generation of start codons, acquisition of splice sites, refinement of codons, etc., leading to the translation of previously non‐coding genomic regions [66, 67, 68, 69]. Sandmann et al. investigated the evolutionary conservation of over 7000 microproteins [70]. Approximately 90% of these microproteins lack homology with non‐primate mammals and can therefore be considered evolutionarily young. Similar to microproteins, most of the JET‐derived isoforms emerged only around 80 million years ago and are conserved across primates [8, 70]. JETs acquired earlier during vertebrate evolution exhibit higher expression levels than younger JETs, suggesting that accumulated mutations in the TE further strengthen its transcription and increase in frequency [8]. Whilst JETs are, in general, evolutionarily young, they are enriched in more ancient genes (i.e., from old phylostrata). Consistently, ancient genes have co‐evolved with the transcriptional machinery, optimizing their expression efficiency and the use of alternative splicing to gain variability and generate novel isoforms [69, 71]. In addition, JETs are a preferential source among alternative splice sites, suggesting that TEs can play a major role in the generation of novel protein isoforms [8, 72].

We propose that the vast reservoir of cryptic transcripts and proteins expressed in human cells is not transcriptional or translational ‘mistakes’. They rather reflect the emergence of novel ORFs through a trial‐and‐error process for adaptive innovation that reflects ongoing evolution in the current human proteome. While this process generates a large pool of unstable proteins, mostly identified in a sample‐specific manner, it simultaneously supplies a diverse repertoire of peptides for MHC‐I presentation (Figure 2). However, a subpopulation of cryptic proteins with potentially advantageous functions is increasing their expression, stability and has eventually become fixed in the population. A longer isoform of ADARB1 is an example of how an unannotated isoform originated from the exaptation of a TE within its catalytic domain is now annotated as part of the canonical proteome [72]. Given the recent inception of cryptic proteins, many of them are likely undergoing the early stages of evolutionary fixation as part of the process of protein innovation.

## 8 Conclusion

Pervasive translation of the human proteome results in thousands of cryptic proteins, heterogeneous in nature, on which evolution can act. The inverse correlation between protein stability and MHC‐I presentation, together with the idea that proteins gain stability when gaining functions, could suggest that functional cryptic proteins are less efficiently processed for MHC‐I presentation compared to non‐functional proteins. Would we therefore be more likely to observe DRiPs and HLA‐presented peptides derived from younger proteins? How does the bias of cryptic proteins in the immunopeptidome versus the whole‐cell proteome impact immunogenicity and tolerance? Characterizing the biogenesis of cryptic proteins will be instrumental to understand their contribution to immunogenicity, uncover new protein functions, and potentially harness them in novel therapeutics.

## Author Contributions

E.G.B. and Y.A.A. wrote the manuscript. S.A., E.G.B. and Y.A.A. contributed to the manuscript design and participated in the discussions that supported it.

## Conflicts of Interest

Yago A. Arribas and Sebastian Amigorena have filed patent applications for the therapeutic use of JET‐derived peptides (WO 2021/043804, WO 2022/189626, WO 2022/189626, WO 2023/180552 and WO 2022/189639). Sebastian Amigorena is an advisor and shareholder in Mnemo Therapeutics.

## Data Availability

Data sharing not applicable to this article as no datasets were generated or analysed during the current study.
